# Downregulation of membrane type-matrix metalloproteinases in the inflamed or injured central nervous system

**DOI:** 10.1186/1742-2094-4-24

**Published:** 2007-09-20

**Authors:** Henrik Toft-Hansen, Alicia A Babcock, Jason M Millward, Trevor Owens

**Affiliations:** 1 Medical Biotechnology Center, University of Southern Denmark, J.B. Winsløwsvej 25, 5000 Odense C, Denmark; 2 Montreal Neurological Institute, McGill University, 3801 University Street, Montreal, Quebec H3A 2B4, Canada

## Abstract

**Background:**

Matrix metalloproteinases (MMPs) are thought to mediate cellular infiltration in central nervous system (CNS) inflammation by cleaving extracellular matrix proteins associated with the blood-brain barrier. The family of MMPs includes 23 proteinases, including six membrane type-MMPs (MT-MMPs). Leukocyte infiltration is an integral part of the pathogenesis of autoimmune inflammation in the CNS, as occurs in multiple sclerosis and its animal model experimental autoimmune encephalomyelitis (EAE), as well as in the response to brain trauma and injury. We have previously shown that gene expression of the majority of MMPs was upregulated in the spinal cord of SJL mice with severe EAE induced by adoptive transfer of myelin basic protein-reactive T cells, whereas four of the six MT-MMPs (MMP-15, 16, 17 and 24) were downregulated. The two remaining MT-MMPs (MMP-14 and 25) were upregulated in whole tissue.

**Methods:**

We used *in vivo *models of CNS inflammation and injury to study expression of MT-MMP and cytokine mRNA by real-time RT-PCR. Expression was also assessed in microglia sorted from CNS by flow cytometry, and in primary microglia cultures following treatment with IFNγ.

**Results:**

We now confirm the expression pattern of MT-MMPs in the B6 mouse, independent of effects of adjuvant. We further show expression of all the MT-MMPs, except MMP-24, in microglia. Microglia isolated from mice with severe EAE showed statistically significant downregulation of MMP-15, 17 and 25 and lack of increase in levels of other MT-MMPs. Downregulation of MT-MMPs was also apparent following CNS injury. The pattern of regulation of MT-MMPs in neuroinflammation showed no association with expression of the proinflammatory cytokines TNFα, IL-1β, or IFNγ.

**Conclusion:**

CNS inflammation and injury leads to downregulation in expression of the majority of MT-MMPs. Microglia in EAE showed a general downregulation of MT-MMPs, and our findings suggest that MT-MMP levels may inversely correlate with microglial reactivity.

## Background

Matrix metalloproteinases (MMPs) are proteinases implicated in all diseases involving neuroinflammation, including multiple sclerosis [1,2]. MMPs are thought to facilitate cellular infiltration of the central nervous system (CNS) by degrading extracellular proteins present in the neurovascular unit, which is composed of endothelial cells connected by tight junctions, and the glia limitans. Inhibition of MMP activity with broad-spectrum synthetic inhibitors alleviates symptoms of experimental autoimmune encephalomyelitis (EAE), an animal model of multiple sclerosis [3-6].

MT-MMPs are implicated in tumor development by promoting angiogenesis and invasion across basement membranes [7,8]. MT-MMPs are expressed in most cancer types and are in many cases linked to malignant parameters [9,10]. Several studies have demonstrated the importance of MMPs in animal models of cancer, but so far MMP inhibitors not been successful in clinical cancer trials [11]. This is likely because the broad-spectrum inhibitors used failed to specifically target detrimental effects of MMPs, and also inhibited beneficial effects [12]. This illustrates the potential importance of understanding the differences between individual MMPs in greater depth.

There are 23 MMPs identified in mice (including two forms of MMP-1). Of these, six (MMP-14, 15, 16, 17, 24, and 25) are referred to as membrane type-MMPs (MT-MMPs) [13]. MMP-17 and 25 are glycosylphosphatidylinositol (GPI)-anchored whereas the other four MT-MMPs are type 1 transmembrane proteins with short cytoplasmic domains of about 20 amino acids. Like secreted MMPs, MT-MMPs can cleave extracellular matrix molecules, as well as chemokines, cytokines and growth factors [9,14]. MT-MMPs are generally thought to play important regulatory roles because of their ability to cleave substrates in the immediate vicinity of the cell membrane, where the cleaved products can interact with cell-surface receptors. In addition, MT-MMPs are known to cleave and activate secreted MMPs, which was first described for activation of MMP-2 by MMP-14 through interaction with tissue inhibitor of metalloproteinases-2 (TIMP-2) [15,16].

The particular roles and substrate specificities of MT-MMPs have not been described in detail, and the role of MT-MMPs in neuroinflammation is unclear. We previously showed that MT-MMPs are downregulated in spinal cord of mice with adoptively transferred EAE (AT-EAE) at peak disease, except for MMP-14 and MMP-25 which are upregulated [17]. The downregulation of the majority of MT-MMPs contrasts with the general upregulation of secreted MMPs. Similar results were obtained by Weaver et al. in actively induced EAE [18]. The functional consequence of this specific pattern of MT-MMP expression is unknown.

In this study, we describe MT-MMP expression by microglia in EAE. We also investigate the relationship between MT-MMP regulation, leukocyte infiltration, and expression of the proinflammatory cytokines TNFα, IL-1β, and IFNγ in three models of neuroinflammation: EAE, pertussis toxin (PTx)-induced parenchymal CNS infiltration in transgenic (Tg) mice expressing the chemokine CCL2 in the CNS, and after CNS injury induced by a cortical stab lesion.

## Materials and methods

### Mice

Wild-type (WT) SJL/J (SJL) and C57BL/6J (B6) were obtained from The Jackson Laboratory (Bar Harbor, Maine, USA) or from Charles River Canada (St. Constant, Quebec, Canada). Transgenic mice expressing the chemokine CCL2 under the control of a myelin basic protein (MBP) promoter [19] were originally obtained from Bristol-Myers Squibb (New Brunswick, New Jersey) and maintained as colonies at the Montreal Neurological Institute, and at University of Southern Denmark. A colony of IFNγR-/- mice on the SJL/J background was derived from mice generously provided by Dr. David Willenborg (Canberra Hospital, Australia). For EAE, female mice 6–12 weeks old were used. Mice subjected to cortical lesion were females weighing 20–25 grams. Animal breeding, maintenance and all experimental protocols were performed in accordance with Canadian Council for Animal Care guidelines as approved by the McGill University Animal Care Committee, or protocols approved by the Danish Ethical Animal Care Committee

### Actively induced EAE

B6 mice were immunized subcutaneously at the base of the tail with 100 μl of an emulsion containing 100 μg myelin oligodendrocyte glycoprotein (MOG)_35–55 _(Sheldon Biotechnology, Montreal, Quebec, Canada) and 500 μg of *Mycobacterium tuberculosis *H37 RA (Difco, Detroit, Michigan, USA) in Freund's incomplete adjuvant (Difco) and boosted in the flanks 7 days later with the same emulsion. PTx (15 μg/kg) was administered intraperitoneally on day 0 and 2. SJL mice were immunized subcutaneously at the base of the tail with 100 μl of an emulsion containing 100 μg proteolipid protein (PLP)_139–151 _(Sheldon Biotechnology) and 200 μg of *Mycobacterium tuberculosis *H37 RA (Difco) in Freund's incomplete adjuvant (Difco) and boosted in the flanks 7 days later. Mice were weighed and monitored daily for clinical signs of EAE, scored as: 1, flacid tail; 2, hind limb weakness and poor righting ability; 3, one hind limb paralyzed; 4, both hind limbs paralyzed with or without forelimb paralysis; 5, moribund.

### Adoptive transfer EAE

AT-EAE in SJL mice was induced by passive transfer of MBP-reactive T cells as described previously [17]. AT-EAE in B6 mice was induced by passive transfer of MOG_35–55 _reactive T cells. B6 mice were immunized as for active EAE and lymph node cells were cultured in the presence of 10 μg/ml MOG_35–55 _(Sheldon Biotechnology) and 5 U/ml IL-2 (Biosource, Nivelles, Belgium). Otherwise, culture conditions were as described for SJL AT-EAE. 10 × 10^6 ^lymphoblasts were transferred into the tail vein. Recipient mice received 15 μg/kg PTx ip on day 0 and 2 after transfer, and were monitored and scored as described for active EAE.

### Administration of pertussis toxin to CCL2 Tg mice

PTx-induced parenchymal CNS infiltration in CCL2 Tg has been described previously [20]. Briefly, PTx (10 μg/kg) (Sigma, Oakville, Ontario, Canada) in Hank's balanced salt solution (Invitrogen Life Technologies, Carlsbad, California, USA) was injected intraperitoneally at day 0. Mice were monitored and weighed daily until sacrifice at day 5.

### Cortical lesion

Under anesthesia, B6 mice were placed into a stereotactic apparatus (Kopf, Tujunga, California, USA) for wireknife transection of axons in the entorhinal cortex as previously described [21]. This induces a cortical stab injury. Twenty-four hours following surgery, mice were perfused with phosphate buffered saline, the cortex was dissected, and approximately 2 mm of tissue surrounding the wireknife lesion was collected. For uninjured controls, the entorhinal cortex was dissected from either the contralateral hemisphere of the brain or from unmanipulated mice.

### Flow cytometry and cell isolation

Cells from whole CNS or isolated entorhinal cortex of mice were prepared for flow cytometry as described [17,21]. Flow cytometry data were analyzed using CellQuest Pro software (BD Biosciences, San Jose, California, USA).

### Quantitative real-time RT-PCR

RNA from whole tissue and sorted cells was purified using Trizol RNA isolation reagent (Invitrogen Life Technologies) according to the manufacturer's protocol. RNA (3 μg) from each tissue sample was incubated with M-MLV reverse transcriptase (RT) (Invitrogen Life Technologies) according to the manufacturer's protocol using random hexamer primers. RNA from sorted cells was incubated with SuperScript II RT (Invitrogen Life Technologies) according to the manufacturer's protocol using random hexamer primers. Quantitative real-time PCR was done using ABI Prism 7000 or 7300 Sequence Detection Systems (Applied Biosystems, Foster City, California, USA). Probe and primer sequences for MT-MMPs [22], IFNγ [23], and TNFα and IL-1β [24] were described previously. Expression of 18S rRNA (primers and probes from Applied Biosystems) in cDNA samples diluted 1/1000 was used to control for differences in the extraction and reverse transcription of total RNA. Each reaction was performed in 25 μl with 50% TaqMan 2× PCR Master Mix (Applied Biosystems), 100 nM each of the forward and reverse primer, and 200 nM of probe. Conditions for the PCR were 2 min at 50°C, 10 min at 95°C, and then 40 cycles, each consisting of 15 s at 95°C, and 1 min at 60°C. Arbitrary cDNA values of individual samples were determined using standard curves obtained from a 4-fold serial dilution of a reference cDNA sample. Relative expression values were calculated by dividing the expression level of the target gene by the expression level of 18S rRNA.

### Isolation and culturing of microglia

Female SJL WT or IFNγR-/- mice (8–10 weeks old) were perfused with 20 ml ice-cold phophate buffered saline, then brains and spinal cords were dissected and collected in Hank's balanced salt solution. The tissue was dissociated through a 70 μm cell strainer (BD Biosciences Pharmingen) and suspended in 70% isotonic Percoll (Amersham Biosciences, Baie d'Urfe, Quebec, Canada). Myelin was removed after centrifugation through a Percoll gradient, and cells were collected at the interface between 30 and 37% Percoll. Cells were washed and resuspended in RPMI 1640 (Gibco, Burlington, Ontario, Canada) supplemented with 10% FCS (Sigma), 50 μM β-mercaptoethanol (Sigma), 2 mM L-glutamine (Gibco), 100 U/ml penicillin and 100 μg/ml streptomycin (Gibco). Finally, cells were diluted to 1 × 10^6 ^cells/ml and plated in a 96 well culture plate (Nunc, Roskilde, Denmark) at a density of 2 × 10^5 ^cells per well. Approximately 2.5 × 10^5 ^microglia were obtained per mouse. An aliquot of cells stained for verification of CD45^dim ^CD11b^+ ^microglia, showed that purity was ≥ 94%. Microglia were left overnight to settle and thereafter treated with mouse recombinant IFNγ (Sigma) for 16 hours. The supernatant was removed and microglia were lysed in Trizol for RNA isolation.

### Statistical analysis

One-way ANOVAs with Bonferroni's post test, or two-tailed unpaired t-tests were applied to analyze results using the GraphPad Prism software (GraphPad Software Inc., San Diego, California, USA). p ≤ 0.05 was considered significant. Error bars represent standard error of the mean.

## Results

### MT-MMP expression in EAE

**Figure 1:**
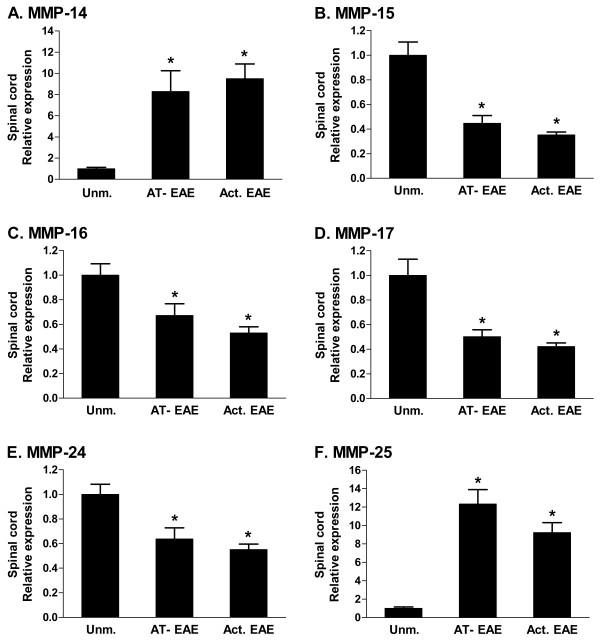
**MT-MMP gene expression in active and AT-EAE in B6 mice**. Gene expression levels of MT-MMPs in spinal cord from unmanipulated B6 mice and mice with severe (grade 4) EAE were measured by real-time PCR. EAE was induced either by immunization with MOG_35–55 _in CFA (active) or adoptive transfer of MOG_35–55 _specific T cells (AT-EAE). Values on the Y-axis are relative to expression of 18S rRNA, and normalized to the expression level of the unmanipulated group. The values are arbitrary and cannot be compared between panels. n = 4 or 5. Unm.: unmanipulated; Act.: active; *: p ≤ 0.05.

We previously found that MMP-15, 16, 17 and 24 are downregulated in spinal cord of SJL mice with MBP-induced AT-EAE, whereas the remaining two MT-MMPs (MMP-14 and 25) are upregulated [17]. The expression of all six MT-MMPs has now been studied in both actively induced and adoptively transferred MOG_35–55 _EAE in B6 mice with severe (grade 4) disease (Fig. 1). Some differences in fold regulation were observed compared to AT-EAE in SJL mice, but statistically significant downregulation of MMP-15, 16, 17 and 24 was observed in both models, as well as upregulation of MMP-14 and 25. Similar results for actively induced EAE in B6 mice have been reported previously [18]. Taken together, this shows that the pattern of MT-MMP regulation in severe EAE is independent of mouse strain, myelin antigen, and use of adjuvant in disease induction.

### Microglial MT-MMP expression in EAE

**Figure 2:**
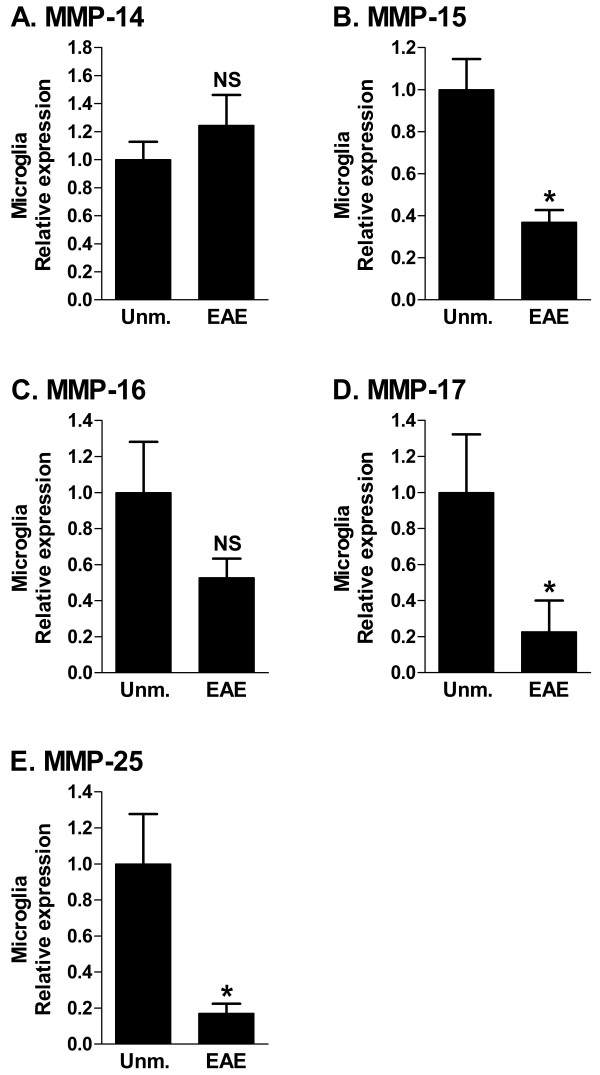
**MT-MMP mRNA expression in microglia sorted from CNS**. Microglia were sorted from whole CNS preparations based on flow cytometric detection of the surface markers CD45^dim ^CD11b^+^. Values on the Y-axis are relative to expression of 18S rRNA, and normalized to the expression level of the unmanipulated microglia. The values are arbitrary and cannot be compared between panels. n = at least 4 in each group; NS: not significant; Unm.: unmanipulated; *: p ≤ 0.05.

Macrophages are known to be major producers of MMPs [17,25]. Microglia share a common myeloid lineage with macrophages, and are considered to be CNS-resident macrophages. Human microglia express a wide spectrum of MMP transcripts, many of which are regulated upon activation *in vitro *[26]. We investigated microglial MT-MMP expression at peak disease (Fig. 2). Microglia were defined as CD45^dim ^CD11b^+ ^cells and sorted using flow cytometry. We observed a statistically significant downregulation of MMP-15, 17 and 25 by microglia in EAE (Fig. 2B, D and 2E) compared to control microglia from unmanipulated mice. This confirms results for MMP-15 published previously [17]. Microglial expression of MMP-14 and 16 (Fig. 2A and 2C) were not significantly altered in EAE. MMP-24 was not expressed by sorted microglia.

### Expression of MT-MMPs is unchanged by PTx-induced parenchymal infiltration in CCL2 Tg mice

To investigate the relationship between the general process of leukocyte infiltration of the CNS, and the pattern of MT-MMP expression, we used a model of neuroinflammation that we have previously described [20]. Leukocytes spontaneously accumulate in the perivascular space in the CNS of transgenic mice which express the chemokine CCL2 under control of a truncated MBP promoter [19]. Systemic administration of PTx causes parenchymal infiltration in CCL2 Tg mice [20]. Expression in brain was not significantly altered for any of the MT-MMPs following administration of PTx to CCL2 Tg mice (data not shown). In contrast to the MT-MMPs investigated in this study, we previously found that secreted MMPs were upregulated upon PTx injection in CCL2 Tg mice [20], suggesting involvement of MMPs in leukocyte migration, while emphasizing the distinction between secreted MMPs and MT-MMPs.

### MMP-15, 17 and 25 are downregulated after CNS stab injury

**Figure 3:**
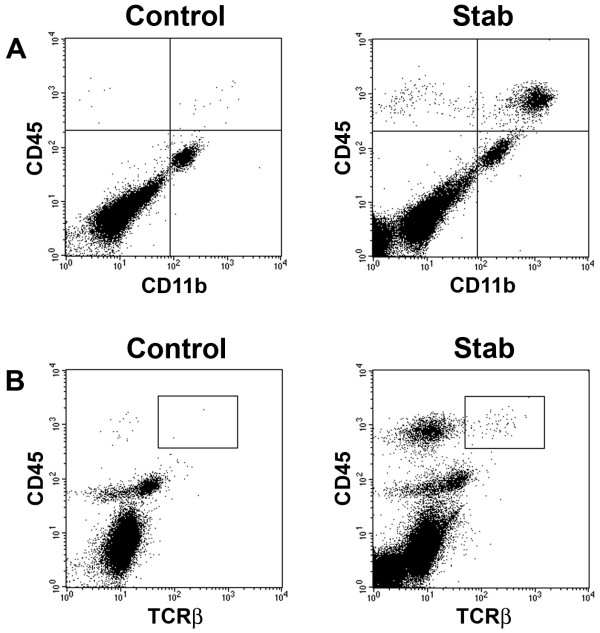
**Flow cytometric analysis of cells in the entorhinal cortex following lesion**. A: Flow cytometry profiles showing CD45^high ^CD11b^+ ^macrophages and granulocytes (upper right quadrants) in uninjured control entorhinal cortex (left panel) and 24 hours after stab injury (right panel) in B6 mice. B: Equivalent analysis of CD45^high ^TCRβ T cells (inserted boxes). The result shown for one mouse in each group is representative of four mice in both groups.

**Figure 4:**
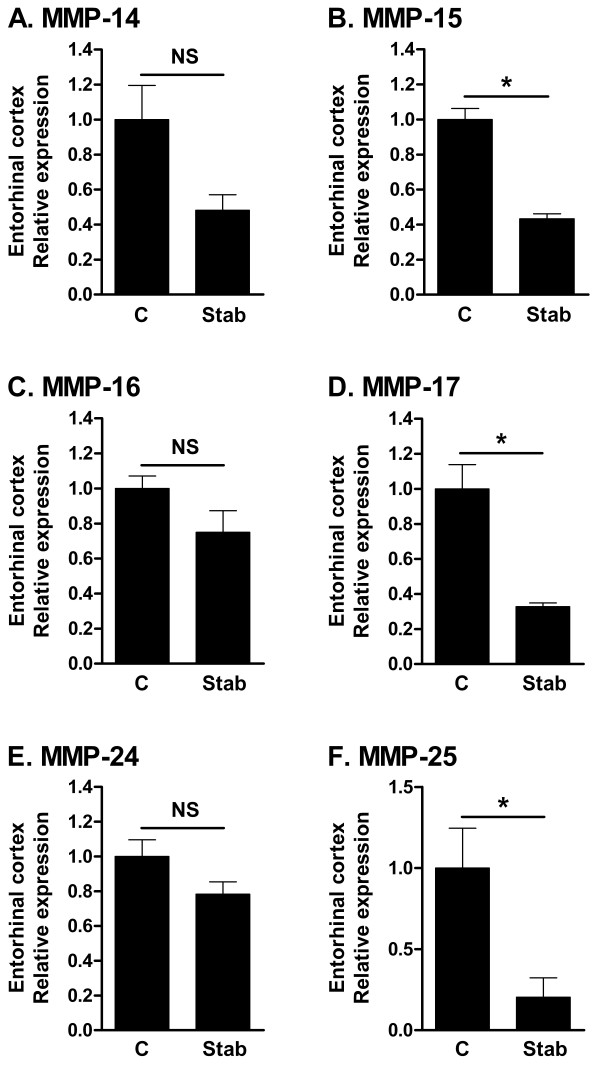
**Gene expression of MT-MMPs after brain stab lesion**. Real-time PCR analysis of MT-MMP expression in entorhinal cortex. Samples were from B6 mice 24 after stab lesion, or from control entorhinal cortex tissue. Values on the Y-axis are relative to expression of 18S rRNA, and normalized to the expression level of the control group. The values are arbitrary and cannot be compared between panels. Significance of comparisons was determined using a two-tailed t-test. n = 6 for C, and 5 for Stab. C: control; NS: not significant; *: p ≤ 0.05.

In contrast to the CCL2 Tg mouse, which represents chemokine-driven leukocyte entry to the CNS, cortical stab injury induces both leukocyte infiltration and glial activation [27,28]. We studied MT-MMP expression following a stab lesion to the entorhinal cortex. Twenty-four hours after stab lesion in the cortex, we observed considerable infiltration of CD45^high ^cells (Fig. 3). The majority of the infiltrating cells were macrophages or granulocytes, defined as CD45^high ^CD11b^+ ^(Fig. 3A, upper right quadrants), and a smaller proportion were T cells defined as CD45^high ^TCRβ^+ ^(Fig. 3B, inserted boxes). MMP-15, 17 and 25 were statistically significantly downregulated following stab lesion, whereas no significant change in expression of MMP-14, 16 and 24 was noted (Fig. 4A–F).

### MT-MMP regulation in neuroinflammation does not relate to expression of the proinflammatory cytokines TNFα, IL-1β, and IFNγ

**Figure 5:**
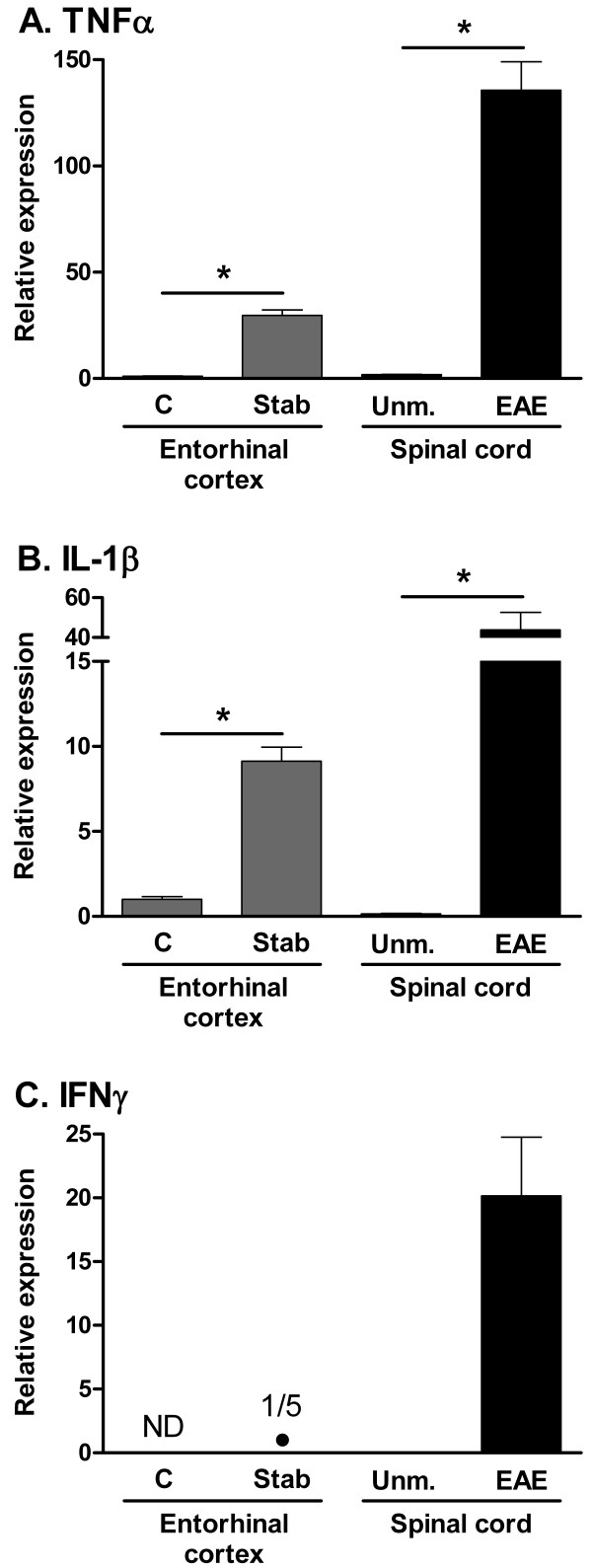
**Gene expression of cytokines after brain stab lesion and in EAE**. Real-time PCR analysis of cytokine expression in entorhinal cortex (left side of graphs) and in spinal cord (right side). Entorhinal cortex samples were from B6 mice 24 after stab lesion, or from control entorhinal cortex. Spinal cord samples were from B6 mice with grade 4 AT-EAE induced by MOG_35–55 _specific T cells, or from unmanipulated controls. IFNγ was only detected in one of 5 stab samples (Panel C). Values on the Y-axis are relative to expression of 18S rRNA, and normalized to the expression level of the control entorhinal cortex group (except for IFNγ). The values are arbitrary and cannot be compared between panels. n = 4–6. ND: not detected; Unm.: unmanipulated; C: control; NS: not significant; *: p ≤ 0.05.

Expression of MT-MMPs can be regulated by growth factors and cytokines [13,29]. We considered the proinflammatory cytokines TNFα, IL-1β, and IFNγ as possible candidates for regulators of MT-MMP expression. Both TNFα and IL-1β were upregulated in EAE and after cortical lesion (Fig. 5), as well as after PTx-induced parenchymal infiltration in CCL2 Tg mice [20]. By contrast, IFNγ, which is absent from unmanipulated CNS, was only upregulated in EAE and not after cortical lesion (Fig. 5C). Unmanipulated CCL2 Tg mice showed a basal level of IFNγ expression in brain, but this was unchanged by systemic administration of PTx (data not shown). Since TNFα and IL-1β showed similar regulation in all three *in vivo *models used, they seemed unlikely candidates to account for the differences seen in MT-MMP expression. We therefore asked whether IFNγ played a regulatory role with respect to MT-MMP expression. This was tested by inducing EAE in IFNγ-/- mice, and assessing MT-MMP expression in the spinal cord using real-time PCR. However, when IFNγ-/- mice with grade 4 EAE were compared to unmanipulated WT mice, we observed the same pattern of MT-MMP expression as in WT mice with grade 4 EAE (data not shown). Thus, absence of IFNγ did not lead to different regulation of MT-MMP expression in EAE spinal cord.

### IFNγ does not regulate microglial MT-MMP expression *in vitro*

**Figure 6:**
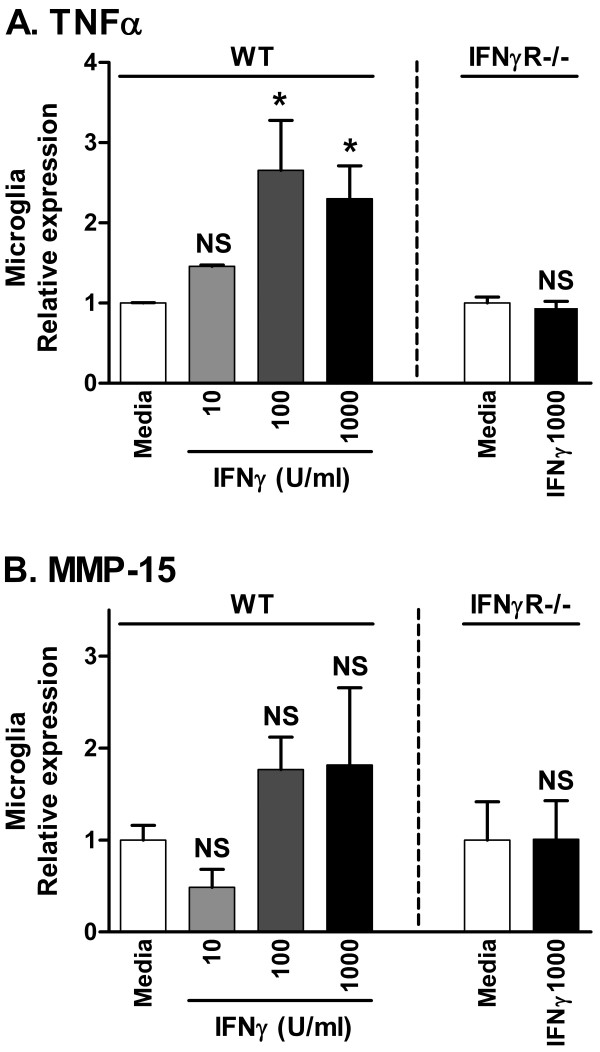
**TNFα and MMP-15 expression by microglia stimulated *in vitro***. Microglia isolated from 12 WT and 8 IFNγR-/- mice were pooled separately and dispersed at 2 × 10^5 ^cells per well. Cultured microglia were stimulated with IFNγ in media at varying concentrations, or media only as control. Each bar graph represents results from three identically treated wells. Values on the Y-axis are relative to expression of 18S rRNA, and normalized to the expression level of the each control group, respectively. The values are arbitrary and cannot be compared between panels. U: units; NS: not significant; *: p ≤ 0.05.

To directly test if IFNγ regulates MT-MMP expression by microglia, we isolated microglia from CNS of adult WT and IFNγR-/- SJL mice and pooled cells from each group. Cell preparations were determined by FACS to contain 94% CD45^dim ^CD11b^+ ^microglia. The microglia were then treated with varying concentrations of recombinant IFNγ. WT microglia responded to IFNγ by upregulating TNFα, whereas IFNγR-/- microglia did not (Fig. 6A). However, no statistically significant change in expression by microglia of MMP-15 (Fig. 6B), or any other MT-MMP (data not shown) was observed in response to IFNγ treatment.

## Discussion

The downregulation of certain MT-MMPs in neuroinflammation does not correspond well with a simple interpretation of MMPs solely as pro-inflammatory mediators. However, given that the specific substrates of individual MT-MMPs remain ill-defined, it is not possible to clearly explain the consequences of the downregulation. One may speculate that microglia downregulate certain MT-MMPs in the process of activation, such as is the case for the innate immune receptor TREM-2 [30,31]. The functional consequence of upregulation of MMP-14, MMP-25 or secreted MMPs in neuroinflammation can be interpreted simply to reflect expression of proteinases by infiltrating cells, enabling them to cross the blood-brain barrier into the CNS by cleaving extracellular matrix proteins. This is most likely not the complete explanation, since MMPs have many other substrates than matrix proteins, and MMPs are likely also involved in regulation of the inflammatory process by cleaving bioactive molecules [14,32].

We confirmed that the previously established pattern of MT-MMP regulation in EAE spinal cord: downregulation of MMP-15, 16, 17 and 24, and upregulation of MMP-14 and 25 is independent of mouse strain, myelin antigen, and use of adjuvant. In microglia sorted from the CNS of mice with EAE, MMP-15, 17 and 25 were downregulated. This corresponds with the change in whole spinal cord for MMP-15 and 17. For MMP-25, the change on the whole spinal cord level was in the opposite direction, which illustrates that changes in gene expression in individual cell types can be masked by changes at the whole tissue level. In this case, the discrepancy is likely due to infiltration of MMP-25 expressing leukocytes. MMP-25 is also known as leukolysin and is expressed by neutrophils [26,33].

Some caution must be exercised in interpreting results that show a small downregulation of mRNA at the whole tissue level in an organ that is undergoing infiltration. We have previously determined the proportion of CD45^high ^infiltrating cells at peak AT-EAE in SJL mice to be 34% in CNS isolates [17]. If such infiltrating cells did not express a particular gene which is expressed by endogenous cells in the unmanipulated CNS, then the influx of these cells alone would lead to an overall downregulation in expression of that gene per cell at the whole tissue level, even though the expression level in individual resident cells did not change. We analyzed microglia from both unmanipulated mice and mice with EAE for direct comparison of this cell type in a disease state with the normal state.

In EAE spinal cord, we found two up and four downregulated MT-MMPs. In the injured entorhinal cortex, we found that MMP-15, 17 and 25 were downregulated, with no change of the remaining MT-MMPs. After PTx-induced parenchymal CNS infiltration in CCL2 Tg mice, we noted no change in MT-MMP expression. The different regulation of MT-MMP expression in these three *in vivo *models of neuroinflammation could potentially be due to different involvement of glial responses and cytokine levels. We investigated whether regulation of proinflammatory cytokines could account for the differences seen in MT-MMP expression. It was previously shown that a stab injury in the cortex lead to induction of IL-1β and TNFα, but not IFNγ[34], and we confirmed this. Comparing the models, TNFα and IL-1β were both significantly upregulated in all three models (Fig. 5A and 5B; and), whereas effects on IFNγ levels differed: IFNγ was absent from unmanipulated CNS, but upregulated in EAE; unmanipulated CCL2 Tg mice showed a basal level of IFNγ, which was unchanged following administration of PTx; and IFNγ was absent, except for a low level in one out of five samples, following cortical lesion. However, *in vivo *experiments in IFNγ-/- mice revealed that absence of IFNγ did not change the regulation of MT-MMP expression in EAE. Likewise, IFNγ did not change MT-MMP expression by microglia *in vitro*.

## Conclusion

We describe a general trend to downregulation of MT-MMPs across *in vivo *models of neuroinflammation. The downregulation of MT-MMPs was generally reflected in microglia, and might be part of an intrinsic component in the microglial response to inflammation and injury. Our findings suggest that downregulation of MT-MMPs occur independently of expression of the proinflammatory cytokines TNFα, IL-1β, and IFNγ.

## List of abbreviations

ANOVA: analysis of variance; AT-EAE: adoptive transfer experimental autoimmune encephalomyelitis; B6: C57BL/6J; CCL: CC chemokine ligand; CNS: central nervous system; EAE: experimental autoimmune encephalomyelitis; IFN: interferon; IL: interleukin; MBP: myelin basic protein; MMP: matrix metalloproteinase; MOG: myelin oligodendrocyte glycoprotein; MT-MMP: membrane type-matrix metalloproteinase; PTx: pertussis toxin; RT: reverse transcriptase; Tg: transgenic; TNF: tumor necrosis factor; WT: wildtype

## Competing interests

The author(s) declare that they have no competing interests.

## Authors' contributions

HT-H and TO conceived the study, and wrote the manuscript. HT-H carried out the experiments and analyzed the results, with the following exceptions: AAB performed entorhinal cortex lesions, and prepared RNA samples from entorhinal cortex. AAB also did flow cytometric analysis of lesioned entorhinal cortex, as well as of cultured microglia. JMM induced EAE in IFNγ-/- mice, and prepared RNA samples from the spinal cords. All authors read and approved the final manuscript.
